# Applications of ultrasound in total synthesis of bioactive natural products: A promising green tool

**DOI:** 10.1016/j.ultsonch.2021.105665

**Published:** 2021-07-18

**Authors:** Sasadhar Majhi

**Affiliations:** Department of Chemistry (UG & PG), Triveni Devi Bhalotia College, Raniganj, West Bengal 713347, India

**Keywords:** Ultrasonic irradiation, Sonochemistry, Total synthesis, Bioactive natural products, Sustainable chemistry

## Abstract

Total synthesis is frequently compared to climbing as it provides a suitable route to reach a high point from the floor, the complex natural product from simple and commercially available materials. The total synthesis has a privileged position of trust in confirming the hypothetical complex structures of natural products despite sophisticated analytical and spectroscopic instrumentation and techniques that are available presently. Moreover, total synthesis is also useful to prepare rare bioactive natural products in the laboratory as several bioactive secondary metabolites are obtained in small quantities from natural sources. The artistic aspect of the total synthesis of bioactive natural products continues to be praised today as it may provide environmental protection through the concept of green or clean chemistry. The use of ultrasound waves as a non-polluting source of energy is of great interest in the field of sustainable and pharmaceutical chemistry as it differs from conventional energy sources in terms of reaction rates, yields, selectivities, and purity of the products. The present review highlights the application of ultrasound as a green tool in the total synthesis of bioactive natural products as well as this article is also aimed to offer an overview of natural sources, structures, and biological activities of the promising natural products for the first time from 2005 to 2020 elegantly.

## Highlights

• Ultrasound has been explored as a green tool in total synthesis.
• The total synthesis of bioactive natural products is grouped into seven parts.
• Natural sources, structures, and biological activities have been highlighted.
• Ultrasound is an eco-friendly, safe, and inexpensive technique.
• Improves reaction rates, yields, selectivities, and purity of the products.

## Graphical abstract

**Figure:**
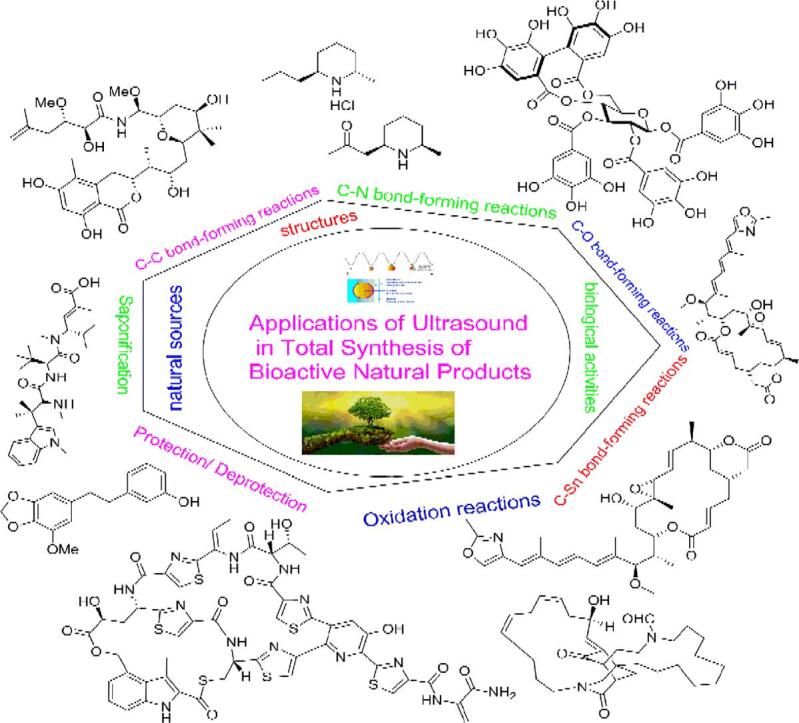

## 1 Introduction

The preparation of urea by Friedrich Wöhler in 1828 gave birth to organic synthesis as well as total synthesis of natural products in the laboratory as total synthesis of bioactive natural products allows step-by-step guidelines for preparing the most promising bioactive agents [1], [2]. With the increasing power of organic synthesis, more and more complex bioactive natural products have been reproduced in the laboratory to confirm the hypothetical most complex structures of bioactive natural products [3], [4]. The field of total synthesis has earned momentum in recent years as the demand for rare bioactive natural products and their derivatives is enhancing because of their use in the area of biology and drug discovery and development. Several bioactive natural products are obtained in small quantities from natural sources, especially those from higher plants and marine organisms [4], despite a magnificent number of modern drugs have been achieved from natural sources [5], [6], [7], [8]. So the development of useful synthetic routes for the total synthesis of most complex bioactive natural products is still a challenging task for synthetic organic chemists [9].

Chemistry, particularly organic chemistry, has a profound role to produce essential materials such as dyes, polymers, cosmetics, agrochemicals, medicines, and many others [10]. Environmental difficulties and unwanted side effects arise due to such types of developments. To protect our Mother Nature we need environmentally benign processes. With the advent of the 21st century, the public is equally aware of the hazardous materials employed and generated by chemical methods, and eventually, the concept and philosophy of green or clean chemistry have evolved [11], [12]. Green chemistry is related to creativity and the development of innovative research. Sustainable chemistry chooses less toxic materials over more toxic ones and tries to minimize the use of flammable, explosive, or highly reactive materials [13], [14]. It has been extensively investigated that the study of chemistry is related to the interaction of energy with matter. Energy is essential for chemical transformations. Thus, this energy to an organic synthesis as well as a total synthesis of secondary metabolites can be supplied by ultrasound as a non-contaminating source of energy. Ultrasound is a unique technique related to cavitation (extreme high local temperatures (around 5000 K) and pressures (over 1000 atmospheres) generated in a liquid phase) which is nowadays a well-regarded eco-environmental technology in organic synthesis and total synthesis of bioactive natural products being advantageous over the classical thermal procedures since enhanced reaction rates, creation of purer products, high yields, enhanced selectivities, easier experimental methods, and use of milder conditions both in case of homogeneous and heterogeneous reactions [15], [16], [17]. Such beneficial qualities as a whole have stimulated the organic chemist and biological community to explore the application of ultrasonic irradiation in more height in the total synthesis of bioactive natural products. The present review focuses on the total synthesis of bioactive natural products by applying ultrasound as a greener technology and the literature covering from 2005 to 2020 for the first time elegantly. In this review, the total synthesis of bioactive natural products by ultrasound are grouped into seven parts such as C–C, C-N, C-O, C-Sn bond-forming reactions, oxidation, protection/deprotection, and saponification reactions and highlights on the natural sources, structures, and biological activities of the promising natural products brilliantly.

## 2 Applications of ultrasound in the total synthesis of bioactive natural products

Ultrasound processing is an eco-friendly, safe, alternative means of activation, and inexpensive technique, employed as a green tool in the field of organic synthesis [16], [17]. This section intends to cover brilliant applications of ultrasound in the total synthesis of bioactive natural products as an unconventional activation technique.

### 2.1 C–C bond-forming reactions under ultrasonic irradiation

Carbon-carbon bond forming reaction is the pillar to construct the carbon skeleton of organic molecules, and therefore it stays at the heart of the chemical sciences and is regarded as the crucial conversion in organic synthesis including the total synthesis of natural products or pharmacologically important compounds to set up the carbon backbone of organic molecules [18]. This section deals with carbon–carbon bond-forming reactions occurring in various bioactive natural products under ultrasound irradiation as a non-polluting source of energy.

#### 2.1.1 Barbier-type reaction under ultrasonic irradiation

##### 2.1.1.1 Total synthesis of psymberin

**Scheme 1:**
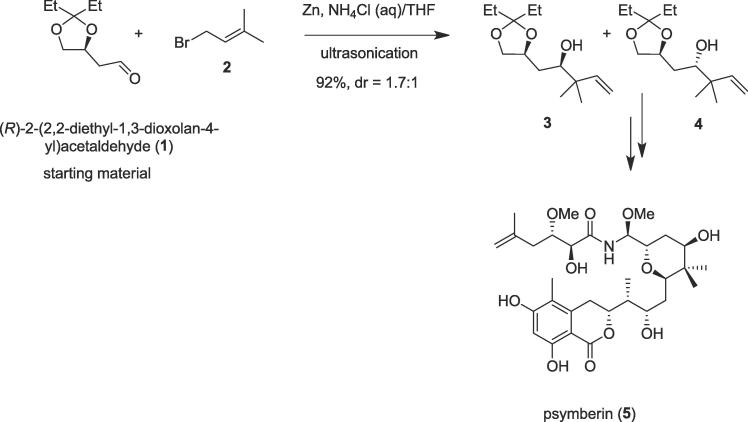
Ultrasound-assisted total synthesis of psymberin.

The Barbier-type reaction is a powerful tool to construct a carbon–carbon bond, as evidenced by the total synthesis of various complex natural products [19], [20]. The Barbier reaction includes the synthesis of primary, secondary, or tertiary alcohol by the treatment of a carbonyl compound with an alkyl halide (chloride, bromide, iodide) in the presence of magnesium, zinc, aluminum, tin, indium, or its salts. This widely utilized reaction bears a close similarity to the Grignard reaction but the key difference is that the organometallic species in the Barbier reaction is created *in situ*, whereas a Grignard reagent is synthesized separately before the addition of the carbonyl compound [21]. Since relatively inexpensive, water insensitive metals or metal compounds are involved in the Barbier reaction so it is possible in several cases to run the reaction in water, making the method part of sustainable chemistry. In 2019, a convergent, stereocontrolled total synthesis of natural psymberin (**5**) was accomplished by Ye and co-workers from known (*R*)-2-(2,2-diethyl-1,3-dioxolan-4-yl)acetaldehyde (**1**) involving Barbier-type reaction under ultrasonic irradiation as a non-polluting tool [22]. A cytotoxic polyketide psymberin (**5**) was isolated from the marine sponge *Psammocinia* sp. obtained from the waters of Papua New Guinea [23], [24]. The synthetic community is motivated to synthesize psymberin (**5**) due to its complex structure possessing 5*S*,8*S*,9*S*,11*R*,13*R*,15*S*,16*R*,17*R* stereochemistry, and interesting biological activity namely antitumor activity [22], [25]. Herein, ultrasound sonication plays an important role to construct a new C–C bond during the Barbier-type reaction. Thus, the known starting aldehyde (**1**) reacted with prenyl bromide (**2**) in the presence of the freshly activated zinc powder under ultrasound sonication to provide a separable mixture (1.7:1) of (**3**) and (**4**) in 92% yield (Scheme 1) [26]. Finally, a highly convergent strategy was executed to complete the total synthesis of bioactive psymberin through ultrasonication as a green tool in 27 steps from starting material using Barbier reaction as a key step.

#### 2.1.2 Blaise reaction under ultrasonic irradiation

##### 2.1.2.1 Total syntheses of (+) - and (–)-dihydrokavain

**Scheme 2:**
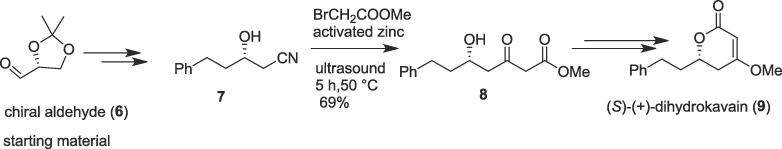
Ultrasound-assisted total synthesis of (*S*)-(+)-dihydrokavain.

The Blaise reaction is an important reaction that occurs between a nitrile and an α-bromoester in the presence of the zinc to give a β-ketoester [27]. This reaction is very useful to create a new C–C bond and it includes good functional group tolerance. For this reason, this reaction has been employed in the preparation of biologically active products [28], [29], [30]; the use of nitriles as electrophiles to produce potentially useful β-ketoesters in the field of medicinal chemistry [31], [32]. Yue et al. accomplished a total synthesis of natural (*S*)-(+)-dihydrokavain (**9**) from the chiral aldehyde (**6**) *via* a green methodology that includes a sonochemical Blaise reaction as the key step [33]. (*S*)-(+)-dihydrokavain (**9**) was isolated from the kava plant (*Piper methysticum*, a Polynesian shrub); this kavalactone (**9**) has been exhibited to be an inhibitor of TNF-α formation and could be effective for the treatment of tumor necrosis factor-alpha (TNF-α) related diseases [34]. Herein, ultrasound plays an important role to produce β-keto ester (**8**) from the nitrile (**7**) *via* Blaise reaction; ultrasound precursor (**7**) was obtained in five steps from the starting material 2,3-*O*-isopropylidene-D-glyceraldehyde (**6**) as a chiral material, easily available from D-mannitol on a large scale [33]. The nitrile (**7**) in tetrahydrofuran (THF) undergoes the Blaise reaction with methyl bromoacetate (BrCH_2_COOMe) in the presence of the activated zinc to furnish δ-hydroxy-β-oxo ester (**8**) in a yield of 69% under ultrasonic irradiation as a green tool at 50 ^0^C for 10 min (Scheme 2). The authors carried out the Blaise reaction as a key step under ultrasonication using BrCH_2_COOMe instead of ethyl bromoacetate (BrCH_2_COOEt) because the latter ester has a very similar *R*_f_ value to that of nitrile (**7**) in TLC, and this made monitoring the conversion more difficult [35]. Finally, the desired bioactive natural product (**9**) was synthesized in eight steps with an overall yield of 25% and its enantiomer (*R*)-(–)-dihydrokavain with an overall yield of 20% in ten steps from the starting material (**6**) through a sonochemical Blaise reaction as an alternative means of activation.

#### 2.1.3 Cascade reaction under ultrasonic irradiation

##### 2.1.3.1 Total syntheses of khayasin, proceranolide, and mexicanolide

**Scheme 3:**
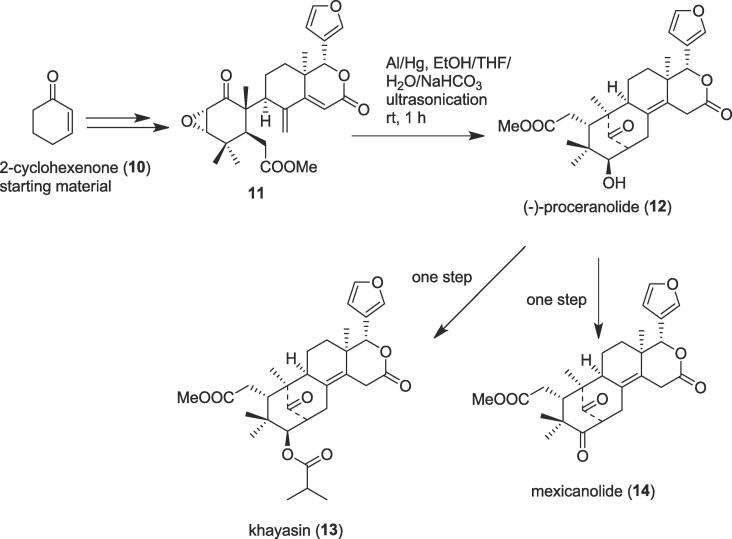
Ultrasound-assisted total syntheses of khayasin, proceranolide, and mexicanolide.

The key steps in the diligent total synthesis of complex natural products include cascade reactions or domino reactions [36], [37]. In these reactions, numerous transformations (at least two consecutive reactions) take place in a single sequence. Cascade reactions are environmentally friendly methods due to their one-pot procedure which accordingly goes ahead in a single reaction solvent, work-up, and purification step and involves a high atom economy [38], [39]. Thus, the first enantioselective total synthesis of the limonoids khayasin (**13**), proceranolide (1**2**), and mexicanolide (**14**) were accomplished by Williams and co-workers in 2012 involving a cascade reaction under ultrasonication as a green tool [40]. The tetranortriterpenoid khayasin (**13**) was isolated from *Khaya senegalensis* [41]; this natural limonoid was found to show a potent and selective insecticide [38], [39], [40] against the devastating Coconut leaf beetle *Brontispa longissima* [42]. The authors began their total syntheses of limonoids from 2-cyclohexenone (**10**) to generate an important intermediate epoxide (**11**) over several steps. Herein, the role of ultrasound was crucial to open the epoxide ring of the intermediate epoxide (**11**) and followed by a 6-*endo-trig* cyclization to furnish the natural limonoid proceranolide (**12**) *via* cascade reaction [43]. Treatment of the vital intermediate (**11)** with freshly amalgamated aluminum pieces in the presence of EtOH/H_2_O/THF/saturated NaHCO_3_ (87:48:30:3 v/v, 1 mL) under ultrasonic irradiation at room temperature (rt) (Scheme 3) provided the desired proceranolide (**12**). Finally, the oxidation of the proceranolide (**12**) in the presence of the Jones reagent yielded natural mexicanolide (**14**) in 68% yield and acylation of the proceranolide (**12**) furnished another natural limonoid khayasin (**13**) in 71% yield.

#### 2.1.4 Cyclization reaction under ultrasonic irradiation

##### 2.1.4.1 Total syntheses of schilancitrilactone B and schilancitrilactone C

**Scheme 4:**
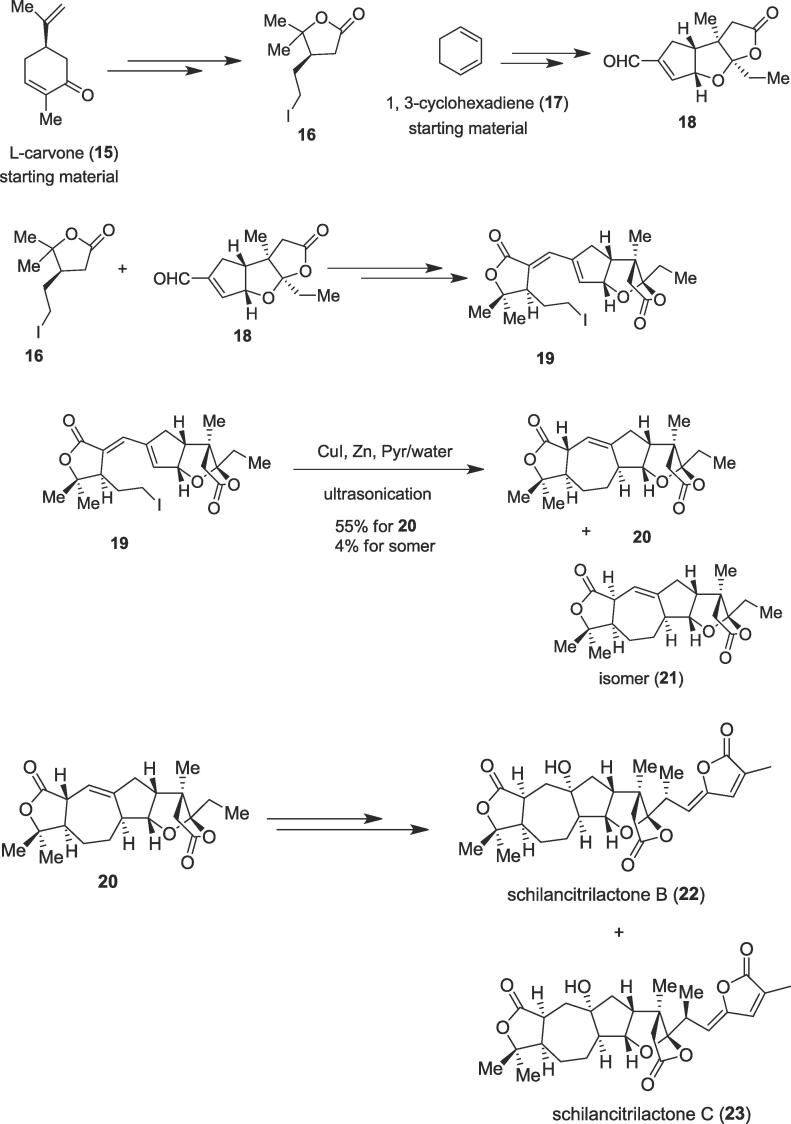
Ultrasound-assisted total syntheses of schilancitrilactone B and schilancitrilactone C.

The intramolecular cyclization method has recently attracted much attention due to its high efficiency, atom economy, and operational simplicity for the total synthesis of medicinally important compounds and bioactive natural products [44], [45], [46], [47]. In 2015, the first total syntheses of schilancitrilactone B (**22**) and schilancitrilactone C (**23**) were achieved by Tang et al. from commercially available materials involving intramolecular cyclization under ultrasonic irradiation as a key step [48]. Two terpenoids schilancitrilactone B (**22**) and schilancitrilactone C (**23**) were isolated from the stems of *Schisandra Lancifolia*, which have been employed in traditional Chinese medicine for the treatment of neurasthenia and related diseases [49], [50]. Highly oxygenated terpenoids schilancitrilactone B (**22**) and schilancitrilactone C (**23**) contain a 5/7/5/5/5-fused pentacyclic ring system carrying nine stereogenic centers and the three *cis*-fused five-membered rings form a structurally rigid tricyclic ring system. The total syntheses of two terpenoids (**22** & **23**) were of very interest to synthetic chemists due to their intriguing structures and biological activities including the anti-human Immunodeficiency Virus (HIV)-1 activity of schilancitrilactone C (**23**) [49], [50]. Their work started with commercially available compounds to afford two important building blocks namely L-carvone (**15**) produced building block (**16**) and another starting material 1,3-cyclohexadiene (**17**) gave another building block (**18**). Next, the iodo compound (**16**) reacted with key intermediate tricyclo lactone (**18**) to furnish ultrasound precursor diene lactone (**19**) in 83% yield over two steps. Next, lactone (**19)** was treated with CuI in the presence of Zn under ultrasonication as an alternative energy input to produce the desired seven-membered cyclization product (**20)** through intramolecular cyclization in 55% yields along with its isomer (**21**) in 4% yield [51] (Scheme 4). The cyclization product (**20)** was able to produce two natural products schilancitrilactone B (**22**) and schilancitrilactone C (**23**) finally; 17 steps (longest linear sequence) were required from starting materials to produce bioactive natural products schilancitrilactone B (**22**) and schilancitrilactone C (**23**) that open a pathway for the syntheses of other compounds linked to these terpenoids, as well as their derivatives.

#### 2.1.5 Cycloaddition reaction under ultrasonic irradiation

##### 2.1.5.1 Total syntheses of (±)-geigerin, (±)- geigerin acetate, and (±)-6-deoxygeigerin

**Scheme 5:**
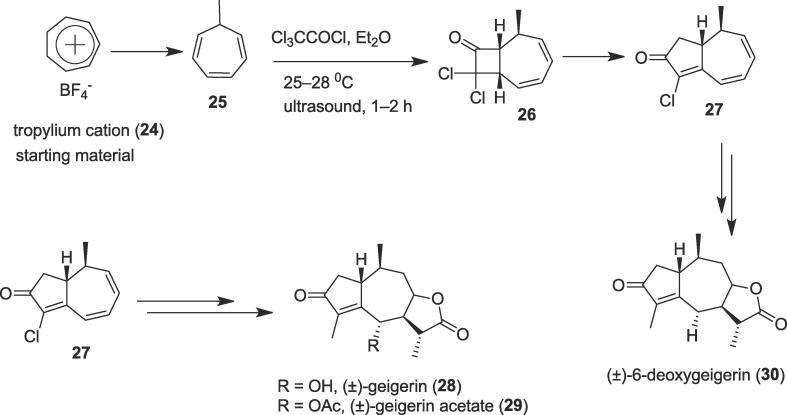
Ultrasound-assisted total syntheses of (±)-geigerin, (±)-geigerin acetate, and (±)-6-deoxygeigerin.

Within the realm of total synthesis of complex natural products, cycloaddition methodology has played a role in ever-increasing importance due to their high atom efficiency. Popular cycloaddition reactions such as Diels-Alder reaction, Paterno Buchi reaction, etc. are 100% atom efficient and cycloaddition reactions have a profound role in the synthesis of bioactive natural products as a green methodology [52], [53], [54]. An efficient stereocontrolled first total synthesis of a guaianolide (±)-geigerin (**28**) was completed by Depres et al. from commercially available tropylium cation (**24**) through cycloaddition reaction under ultrasonic irradiation [55]. Guaianolides, which contains a bicyclo[5.3.0]decane skeleton, a large group of naturally occurring sesquiterpene lactones [56]. Guaian-6,12-olides and guaian-8,12-olides are two forms of guaianolides; they exhibit important, diverse biological activities [57]. Geigerin (**28**) contains six stereogenic centers which are a member of the guaian-8,12-olide class; it was isolated from *G. aspera Harv*., a South African species of *Geigeria*, called colloquially as the vermeerbos (“vomiting bush”) and it shows cytotoxicity [58]. A versatile intermediate [59] hydroazulenone (**27**) was obtained from the starting material (**24**) over three steps in 43% overall yield through a highly regio- and stereocontrolled [2 + 2] cycloaddition/ring-expansion/ elimination sequence [60] using ultrasound as a non-polluting source of energy. Ultrasound precursor 7-methylcyclohepta-1,3,5-triene (**25**) was treated with trichloroacetyl chloride (Cl_3_CCOCl) in Et_2_O at 25–28 ^0^C for 1–2 h to furnish the key intermediate hydroazulenone (**27**) through the formation of the bicyclo keto compound (**26**) under ultrasonication as a green tool [Scheme 5]. Finally, hydroazulenone (**27**) afforded (±)-6-deoxygeigerin (**30**) in four steps which represent a highly efficient second-generation total synthesis; the first total synthesis of (±)-geigerin (**28**) (easily converted to (±)- geigerin acetate (**29**) in only eight steps (4.9% overall yield) from the starting material was also achieved without the need of protecting groups as an another green methodology.

#### 2.1.6 Cyclopropanation reaction under ultrasonic irradiation

##### 2.1.6.1 Total syntheses of aeruginosin 98B and 298A

**Scheme 6:**
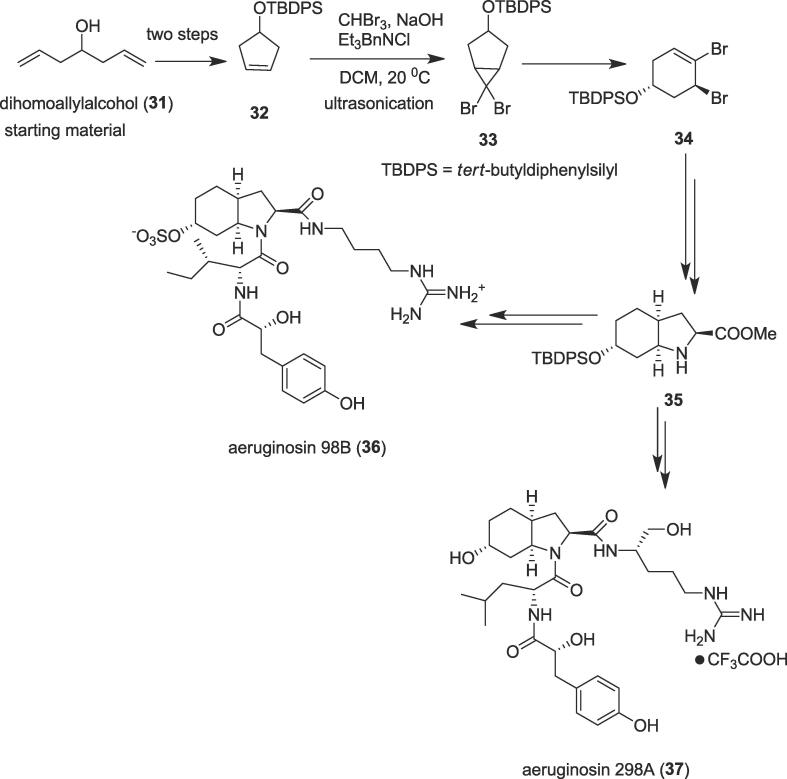
Ultrasound-assisted total syntheses of aeruginosin 98B and 298A.

Cyclopropanes, one of the most important strained rings, have earned much attention for more than a century due to their attractive and unique reactivity. They not only be present in many bioactive natural products and pharmaceutical molecules including terpenes, pheromones, fatty acid metabolites, and unusual amino acids but have also been widely employed in the area of organic synthesis, medicinal chemistry, and materials science as multipurpose building blocks [61], [62], [63]. In 2015, an efficient and scalable synthesis of aeruginosin marine natural products was explored by Baudoin et al. based on cyclopropanation reaction under ultrasonication as a more efficient source of heating [64]. Aeruginosins including aeruginosin 98B (**36**) and 298A (**37**) were isolated from marine sponges and cyanobacterial water blooms; they show potent inhibitory activity against serine proteases [65], [66]. Structurally the aeruginosins 98B (**36**) and 298A (**37**) comprise a 2-carboxy-6-hydroxyoctahydroindole (Choi) core (**35**) and a D-hydroxyphenyllactic (Hpla) subunit. The ultrasonic irradiation and the first C(sp^3^)-H activation reaction were employed to construct hydroxyoctahydroindole (Choi) core (**35**) and the second C(sp^3^)-H activation reaction was useful for the formation of the D-hydroxyphenyllactic (Hpla) subunit. The total syntheses of the aeruginosins 98B (**36**) and 298A (**37**) were commenced from the commercially available dihomoallylalcohol (**31**) to afford ultrasound precursor the cyclopentenol (**32**) in quantitative yield over two steps. Treatment of the cyclopentenol (**32**) with bromoform and NaOH in the presence of the benzyltriethylammonium chloride (Et_3_BnNCl) in dichloromethane (DCM) under ultrasonic irradiation at 20 ^0^C provided dibromobicyclo diphenylsilane (**33**) (Scheme 6). In this vital step, bromoform reacted with NaOH to produce dibromo carbene intermediate which added to the double bond of the cyclopentenol (**32**) under ultrasonication as a green synthetic tool *via* cyclopropanation reaction followed by thermal electrocyclic ring-opening [67] to furnish the racemic dibromocyclohexene (**34**) as an inconsequential 6:1 mixture of diastereoisomers. Ultimately, the 2-carboxy-6-hydroxyoctahydroindole (Choi) core (**35**) provided secondary metabolites aeruginosins 98B (**36**) and 298A (**37**) successfully.

#### 2.1.7 Michael addition under ultrasonic irradiation

##### 2.1.7.1 Total synthesis of (-)-stenine

**Scheme 7:**
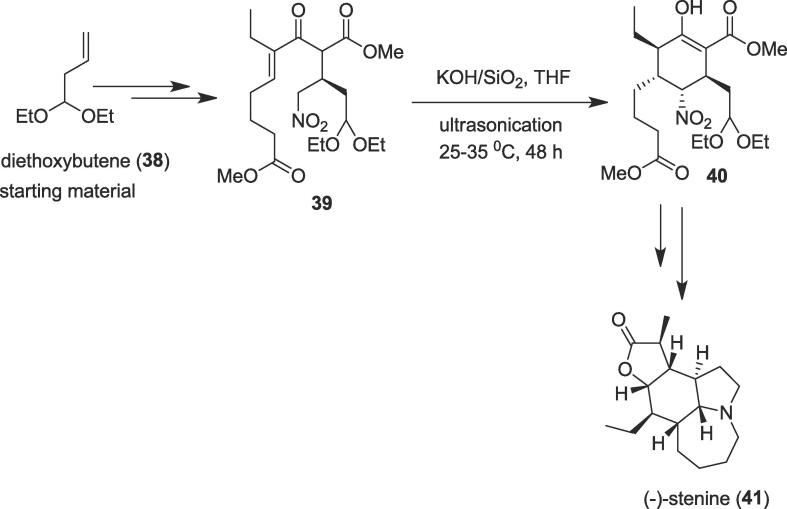
Ultrasound-assisted total synthesis of (-)-stenine.

The Michael addition is the classical 1,4-addition of a nucleophile to an α,βunsaturated system. It is one of the most important reactions for the formation of carbon–carbon bonds and this reaction is widely employed to synthesize all kinds of natural products and drugs. The Michael addition as well as intramolecular Michael addition with great efficiency, simplicity, and greenness has been the goal pursued by the organic synthetic community [68], [69], [70]. Zhang and co-workers disclosed an efficient enantioselective total synthesis of (-)-stenine (**41**) using ultrasound as a non-polluting source of energy for the intramolecular Michael addition with high diastereoselectivity in 2013 [71]. This unique *Stemona* alkaloid was isolated from the roots of *Stemona* species; it contains a pyrrolo[*1,2*- *a*]azepine nucleus and a densely substituted perhydroindole ring system as well as the seven contiguous stereogenic centers [72], [73] which is a challenge for the asymmetric organic synthetic community. Alzheimer's Disease (AD) is the most general type of dementia in present society and had an intense economic and social impact as the aging of populations continuing [74]. Now, the most extensively accepted biochemical theory of the disease, called the cholinergic hypothesis, is that the decline in cognitive and mental functions related to AD is linked to the loss of cortical cholinergic neurotransmission. Acetylcholinesterase (AChE, EC3.1.1.7) plays an important role at the end of nerve impulse transmission at the cholinergic synapses by quick hydrolysis of acetylcholine (ACh). It has been evaluated that stenine B and stenine exhibited anti-acetylcholinesterase activities, with the half-maximal inhibitory concentration (IC_50_) values of 2.1 ± 0.2 μM and IC_50_ = 19.8 μM respectively [74]. Hence, the authors commenced their total synthesis of (-)-stenine (**41**) from commercially available diethoxybutene (**38**) to afford a valuable intermediate α,β-unsaturated keto ester (**39**) over several stages. Next, α,β-unsaturated keto ester (**39**) undergoes intramolecular Michael addition under ultrasonic irradiation to yield Michael addition product (**40**) as a key intermediate in 80% yield together with β-ketoester in 11% yield (Scheme 7). The intramolecular Michael addition was completed in the presence of potassium hydroxide (KOH) that was supported on silica gel (KOH/SiO_2_) in anhydrous THF with ultrasound. The cyclization occurred with high diastereoselectivity in the presence of the KOH/SiO_2_. Finally, the key intermediate (**40**) provided the desired natural alkaloid (-)-stenine (**41**) in 14 steps from commercially available material in an overall yield of 5.9%.

#### 2.1.8 Reformatsky-Claisen rearrangement under ultrasonic irradiation

##### 2.1.8.1 Total syntheses of (-)-dihydrosporothriolide and 3-epi-dihydrosporothriolide

**Scheme 8:**
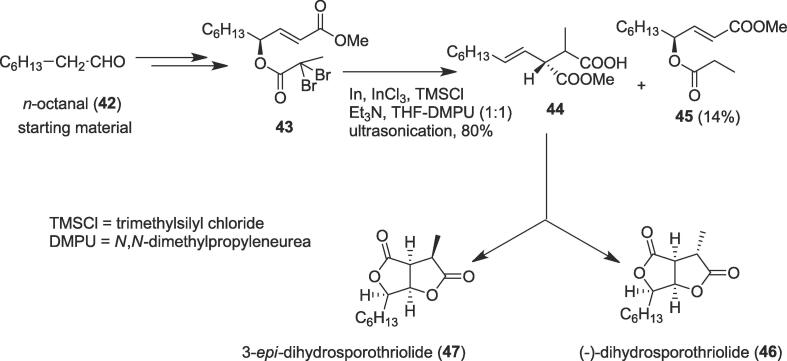
Ultrasound-assisted total syntheses of (-)-dihydrosporothriolide and 3-*epi*-dihydrosporothriolide.

The [3], [3]-sigmatropic Claisen rearrangement is one of the most powerful methods to provide useful building blocks for the synthesis of natural products. The In-mediated Reformatsky-Claisen rearrangement has been used for the formation of the new carbon–carbon bond with high atom economy [75], [76]. In 2014, Hatakeyama et al. planed to synthesize (-)-dihydrosporothriolide (**46**) to clarify the structural ambiguity as the spectral data of this natural dihydrosporothriolide were quite different from those reported for the synthetic sample. They also completed the total synthesis of 3-*epi*-dihydrosporothriolide (**47**) through the Reformatsky-Claisen rearrangement as a key step under ultrasonic irradiation [77]. Dihydrosporothriolide (**46**), a biologically active bis-γ-butyrolactone, was isolated from *Xylaria* sp [78]; the fused bis-γ-butyrolactones have interested considerable attention in the organic synthetic and biological communities due to their extremely oxygenated compact structures and magnificent biological properties. For instance, avenaciolide shows remarkable antifungal and antibacterial activities [79], sporothriolide exhibits significant antibacterial, fungicidal, algicidal, and herbicidal activities and its hydrogenated product, namely, 3-*epi*-dihydrosporothriolide (**47**), bears significant antibacterial and herbicidal activities [80]. The concise asymmetric total synthesis of natural (-)-dihydrosporothriolide (**46**) was initiated from *n*-octanal (**42**) to furnish an important intermediate α,α-dibromopropionate (**43**). An interesting Reformatsky-Claisen rearrangement of the α,α-dibromopropionate (**43**) took place smoothly to afford a 2:1 mixture of carboxylic acids (**44)** in 80% yield, together with debrominated product (**45**) in 14% yield under ultrasonic irradiation. Treatment of dibromo unsaturated ester (**43**) with In and Indium(III) chloride (InCl_3_) in the presence of the trimethylsilyl chloride (TMSCl) and Et_3_N in THF-DMPU (*N*,*N′*-dimethylpropyleneurea) (1:1) provided the rearranged product (**44**) in 80 % yield (Scheme 8). Finally, the rearranged product (**44**) was able to synthesize natural (-)-dihydrosporothriolide (**46**) and its epimer 3-*epi*-dihydrosporothriolide (**47**) successfully and the total synthesis completed in seven steps in 17% overall yield from starting material.

### 2.2 C-N bond-forming reaction under ultrasonic irradiation

C-N bonds are omnipresent in natural products, pharmaceuticals, and biologically active compounds. Nitrogen-containing molecules are of great importance because of their attractive and diverse biological properties. Despite significant developments in this area, the construction of the C-N bond is still a major challenge for the organic synthetic community, because of the involvement of harsh reaction conditions or the need for expensive catalysts in several cases. Therefore, it is important to develop synthetic methods that both effectively build C-N bonds and incorporate principles of green chemistry. These principles involve the use of renewable energies sources such as ultrasonic irradiation, sunlight and enhancing the atom economy of the transformations [81], [82]

#### 2.2.1 Total syntheses of (+)-dihydropinidine, (-)-epi-dihydropinidine, and (-)- pinidinone

**Scheme 9:**
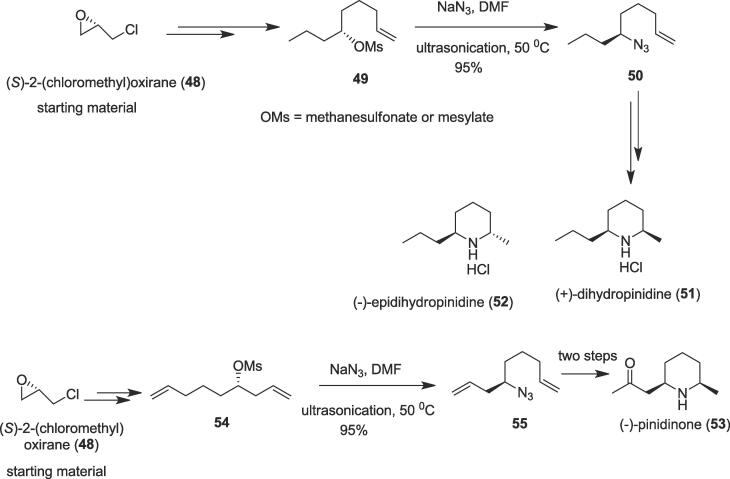
Ultrasound-assisted total syntheses of (+)-dihydropinidine, (-)-epidihydropinidine, and (-)- pinidinone.

The shortest routes for the total syntheses of three naturally occurring alkaloids (**51**–**53**) were developed by Szolcsanyi and et al. in 2011with the highest overall yields (32%-54%) from (*S*)-epichlorohydrin (**48**) as a common substrate using C-N bond formation as key steps under ultrasonic irradiation [83]. The 2,6-disubstituted piperidine alkaloids (+)-dihydropinidine (**51**), (-)-epidihydropinidine (**52**) (as HCl salts), and (-)- pinidinone **(53**) were isolated from many *Picea* (spruce) and/or *Pinus* (pinus) species [84] and various insects [85]. Regarding the biological property, (-)-epidihydropinidine (**52)** was described to have moderate to high antifeedant activity against eastern spruce budworm [84]. The common starting material (*S*)-epichlorohydrin (**48**) was efficiently converted to the mesylate (**49)** over four steps. Herein, ultrasonic irradiation plays a significant role to provide a key substrate alkenylazide (**50**) through the C-N formation. Treatment of the mesylate (**49)** with sodium azide **(**NaN_3_) in dimethylformamide (DMF) under ultrasonic irradiation at 50 ^0^C delivered alkenylazide (**50**) in a 95% yield (Scheme 9); (+)-dihydropinidine (**51**), (-)-epidihydropinidine (**52**) (as HCl salts) alkaloids were also obtained from a common key substrate alkenylazide (**50**). Ultrasonic irradiation was also employed to construct a new C-N bond for the generation of another natural alkaloid (-)- pinidinone **(53**); the intermediate mesylate (**54**) was treated with NaN_3_ in DMF to give the dialkenylazide (**55**) in 95% yield at 50 ^0^C under ultrasonic irradiation as another key step.

### 2.3 C-O bond-forming reaction under ultrasonic irradiation

Carbon-oxygen bond formation reaction is one of the most significant procedures in organic synthesis. The benzo-fused oxygen heterocycles are found as a key structural unit in natural products, medical and pharmaceutical molecules. Plant-derived natural products include phenolic functional groups namely green tea bears catechin compounds like epigallocatechin gallate and the epicatechins having pharmacological activities [86], [87].

#### 2.3.1 Total syntheses of strictinin and tellimagrandin II

**Scheme 10:**
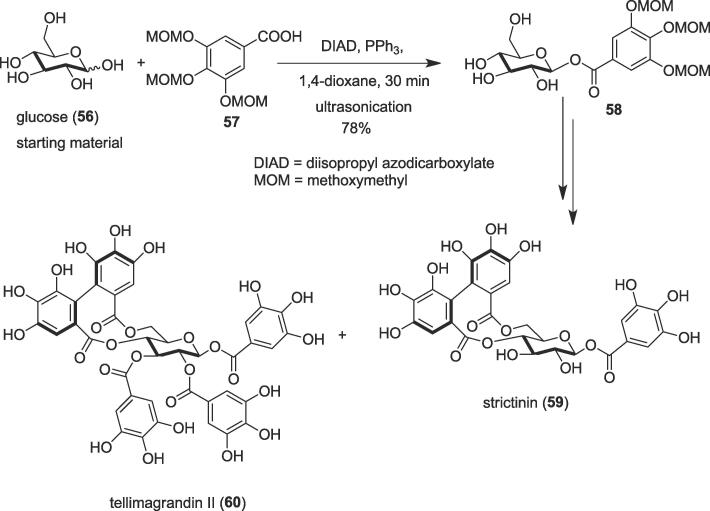
Ultrasound-assisted total syntheses of strictinin and tellimagrandin II.

Short total syntheses of natural glycosides ellagitannins strictinin (**59**) and tellimagrandin II (**60**) were developed by Kawabata and co-workers in 2015 through the sequential and regioselective introduction of galloyl(oxy) groups to unprotected glucose [88]. Glucose was employed as a starting material to synthesize bioactive ellagitannins strictinin (**59**) [91] and tellimagrandin II (**60**) *via* stereoselective glycosidation under ultrasonication as an alternative source of energy. Strictinin (**59**), the main phenolic compound in Pu'er teas originated from young leaves and buds of wild trees [89] and tellimagrandin II was obtained from *Geum japonicum* and *Syzygium aromaticum* [90]. The architecturally intriguing and biologically potent ellagitannins have spurred interest in the organic synthetic community. Structures of strictinin (**59**) and tellimagrandin II (**60**) primarily comprise a central sugar core, typically D-glucose, to which esterified gallic acid and hexahydroxy diphenoic acid groups are attached [88]. Anti-HSV (Herpes simplex virus), antitumor, anti-influenza virus, and antiallergic activities were exhibited by these compounds [91], [92], [93], [94]. Herein, ultrasound sonication was employed to generate an ester linkage as a key step *via* Mitsunobu conditions [95]. Thus, treatment of unprotected glucose (**56**) as a glycosyl donor with gallic acid trimethoxymethyl ether (**57**) in 1,4-dioxane in the presence of the diisopropyl azodicarboxylate and triphenyl phosphine at room temperature for 30 min to furnish the desired product (**58**) with high stereoselectivity (β/α = 99/1) in 78% yield under ultrasound sonication (Scheme 10). The use of ultrasonication and finely ground glucose powder in 1,4-dioxane before the addition of the Mitsunobu reagents were found to be key for the smooth development of glycosylation. Strictinin (**59**) was synthesized from naturally abundant glucose in five overall steps in 21% overall yield and another ellagitannin tellimagrandin II (**60**) was prepared from the same starting material in six overall steps in 18% overall yield.

### 2.4 C-Sn bond-forming reaction under ultrasonic irradiation

C-Sn bond is called a kind of very significant carbon–metal bond, which has many applications in organic chemistry, medicinal chemistry, and biological chemistry [96], [97], [98].

#### 2.4.1 Total synthesis of WF-1360F

**Scheme 11:**
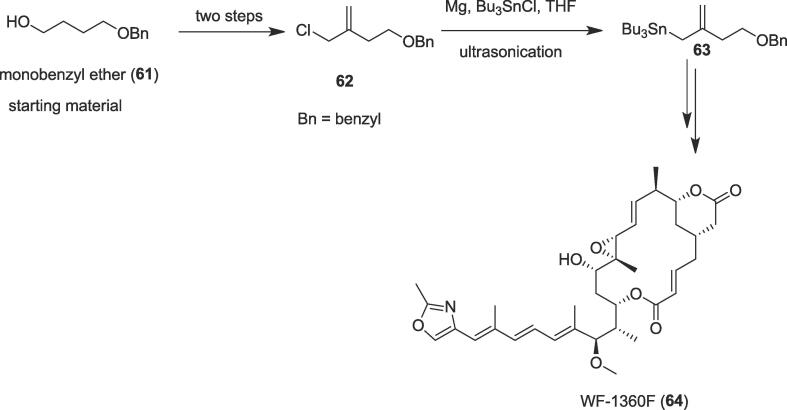
Ultrasound-assisted total synthesis of WF-1360F.

The first total synthesis of the antimitotic WF-1360F (**64**) was explored by Altmann et al. in 2013 through ring-closing alkyne metathesis (RCAM) as an alternative approach to macrocycle formation and ultrasound as an alternative means of activation [99]. Tubulin inhibitor WF-1360F (**64**) was isolated from fermentation broths of *Rhizopus chinensis* [100] and *Burkholderia rhizoxina* [101]; the growth inhibitory activity was exhibited by natural WF-1360F (**64**) [100], [101]. The authors commenced their total synthesis of a 16-membered macrolide WF-1360F (**64**) from mono benzyl ether (**61**) to produce allylic chloride (**62**) over two steps *via* Appel reaction as a key step. The allylic chloride (**62**) undergoes an ultrasound-promoted Barbier-type reaction [102] to furnish stannane (**63**). Treatment of the allylic chloride (**62**) with Mg turnings and tributyltin chloride (Bu_3_SnCl) in THF afforded tin hybride (**63**) through the formation of the new C-Sn bond in quantitative yield under ultrasonication at 0 ^0^C to room temperature (Scheme 11). Ultimately, bioactive natural product WF-1360F (**64**) was synthesized from stannane (**63**) over several steps based on macrocyclic ring-closure and the useful conversion of the ensuing alkyne moiety into the needed *E*-configured double bond. The antiproliferative property of WF-1360F (**64**) was also performed by the same group against the human pancreatic and colon cancer cell lines MiaPaCa and HCT116 and it has been shown that IC_50_ values (5.1 ± 0.74 and 4.5 ± 0.38) of WF-1360F (**64**) were in the single-digit nanomolar range, which is in general consistency with the data suggested for natural macrolide on K-562 and L929 cells [101].

### 2.5 Oxidation under ultrasonic irradiation

Oxidation is a method by which a carbon atom acquires bonds to more electronegative elements, most frequently oxygen. An important direction in the field of organic chemistry is selective oxidation, which gives several synthetically useful intermediates, all of which are widely exploited in total syntheses of natural products [103], [104].

#### 2.5.1 Total syntheses of (-)-dihydrostilbenes

**Scheme 12:**
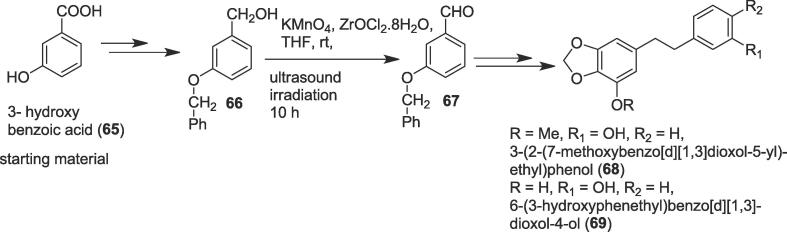
Ultrasound-assisted total syntheses of (-)-dihydrostilbenes.

Yao et al. achieved the first total syntheses of two natural dihydrostilbenes, 3-(2-(7-methoxybenzo[*d*][1,3]dioxol-5-yl)-ethyl)phenol (**68**) and 6-(3-hydroxyphenethyl)benzo[*d*][1,3]- dioxol-4-ol (**69**) in 28% and 20% overall yield respectively with significant anti-proliferative activity against human cancer cell lines [105]. Stilbenoids 3-(2-(7-methoxybenzo[*d*][1,3]dioxol-5-yl)-ethyl)phenol (**68**) and 6-(3-hydroxyphenethyl)benzo[*d*][1,3]-dioxol-4-ol (**69**) were isolated from *Bulbophyllum odoratissimum* Lindl, a folk herb for the treatment of phthisis and rheumatism in the southern part of China [106]. The authors began their work from 3-hydroxy benzoic acid (**65**) to furnish primary alcohol (**66**) in 88.9 % yield over three steps. Herein, the role of ultrasound irradiation was to produce a key aldehyde (**67**) in 98.5 % yield from primary alcohol (**66**) as a non-polluting tool through the oxidation reaction. Treatment of the benzyloxy alcohol (**66**) with KMnO_4_ in the presence of the zirconyl chloride (ZrOCl_2_·8H_2_O) in THF at room temperature for10 h under ultrasound provided the aromatic aldehyde (**67**) (Scheme 12). Finally, the key aldehyde (**67**) was able to prepare the desired two natural dihydrostilbenes (**68** and **69**) *via* Wittig-Horner reaction as another key step. Besides, nine analogues of (**68**) and (**69**) were also synthesized and evaluated for their anti-proliferative property against SGC-7901, KB, and HT-1080 cell lines employing MTT assay [105].

#### 2.5.2 Total synthesis of (-)-gracilioether F

**Scheme 13:**
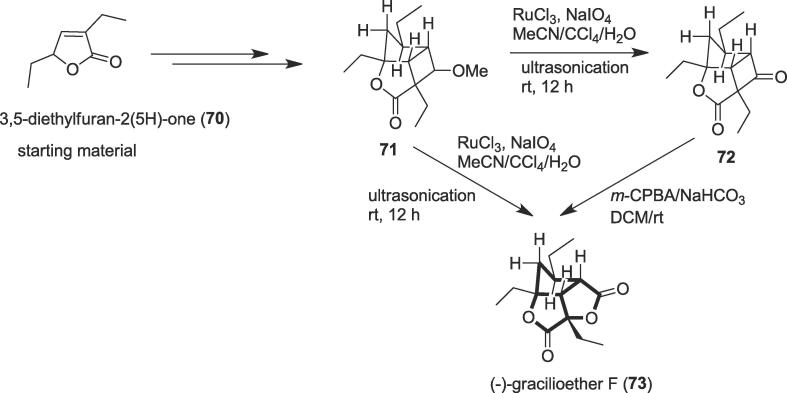
Ultrasound-assisted total synthesis of (-)-gracilioether F.

In 2018, Tang and co-workers disclosed the enantioselective total syntheses of *Plakortin* polyketides, a potent antifungal hippolachnin A, and a series of closely relevant congeners namely gracilioethers with high step-economy and overall efficiency involving oxidation reaction as a key step [107]. The gracilioethers such as (-)-gracilioether F, (+)-gracilioether A, (-)-gracilioether E, etc. are a class of polyketides isolated from various families of marine sponge with rich chemical diversity and potential biological activities [108], [109]. Among these marine sponge-derived natural products gracilioether F (**73**) was isolated from marine sponge *Plakinastrella mamillaris* in 2012 [110], along with gracilioethers A, E, G, H, I, and K were obtained from marine sponges of the genera *Plakinastrella*, *Plakortis,* and *Agelas* as rich sources of gracilioether family [108]. Most gracilioethers bear densely functionalized bridged tricyclic backbone having at least five contiguous stereocenters and a number of these family members demonstrate significant biological activities. For instance, gracilioether A exhibits potential antimalarial activities against *Plasmodium falciparum* (IC_50_ = 10 μg/mL) and gracilioether H shows promising antiplasmodial activity against chloroquine resistant CR FC29 strain (IC_50_ = 3.26 μM) [110], [111] and gracilioethers (E, F, G, H, I, and K) via a reductive cleavage. The tricyclic core which is the structural uniqueness of gracilioether F (**73**) and the dense arrangement of stereogenic centers would give a unique challenge to state-of-the-art chemical synthesis. The enantioselective total synthesis of the (-)-gracilioether F (**73**) commenced from 3,5-diethylfuran-2(5H)-one (**70)** as a starting material to afford the [2 + 2] adduct (**71)** as a single diastereoisomer. Next, the oxidative cleavage of the methyl ether of (**71**) took place to furnish the cyclobutanone (**72**) in a yield of 80% in the presence of the RuCl_3_/NaIO_4_ at room temperature under ultrasonic irradiation as a non-polluting tool [112] (Scheme 13). Finally, the treatment of cyclobutanone (**72**) in dichloromethane (DCM) with *meta*-chloroperoxybenzoic acid (*m*-CPBA) in the presence of NaHCO_3_ provided the desired product (-)-gracilioether F (**73**) in 90% yield at room temperature. In another way, methyl ether (**71**) was also able to produce the desired product (-)-gracilioether F (**73**) directly at room temperature under the same condition through ultrasonic irradiation as a green tool.

### 2.6 Protection/ deprotection under ultrasonic irradiation

A protecting group is a molecular framework that is introduced into a specific functional group in a poly-functional molecule to block its reactivity under multistep reaction conditions. The concept of protection is very helpful for a chemoselective reaction. The deprotection of the functional group occurs to remove the protected functional group to obtain the desired product. Fine chemicals are complex and multifunctional compounds, frequently characterized by low volatility and limited thermal stability, whose manufacture normally is based on multistep synthesis carried out in the liquid phase and often involving protection-deprotection steps [113].

#### 2.6.1 Protection under ultrasonic irradiation

##### 2.6.1.1 Total syntheses of (–)-strictamine, 16-epi-strictamine, and (–)-rhazinoline

**Scheme 14:**
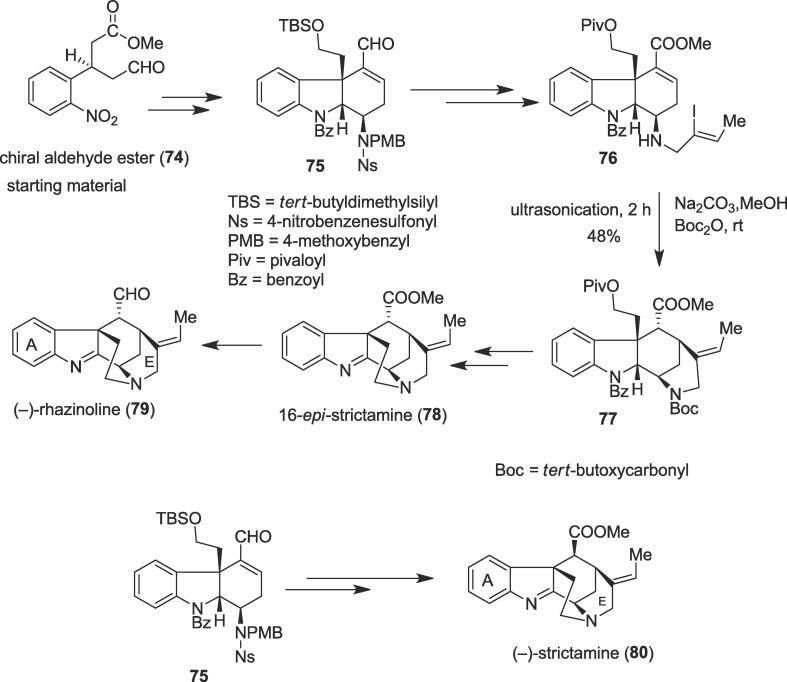
Ultrasound-assisted total syntheses of (–)-strictamine, 16-*epi*-strictamine, and (–)-rhazinoline.

In 2019, a unified approach to the enantioselective total syntheses of (–)-strictamine (**80**), 16-*epi*-strictamine (**78**), and (–)-rhazinoline (**79**) were disclosed by Qin and co-workers involving the protection of the secondary amine under ultrasonication as a key step [114]. Strictamine (**80**) and associated congeners such as rhazinoline (**79**) [115] belong to a subfamily of the akuammiline alkaloids that possess a unique methanoquinolizidine structural unit; they contain different stereochemistry at the C16 position. The akuammiline alkaloids, a monumental group of monoterpenoid indole alkaloids, were isolated from species of the Apocynaceae family eminently [116]; these plant sources and their chemical constituents show a wide range of bioactivities [117]. Strictamine (**80**) and rhazinoline (**79**) akuammiline alkaloids were isolated from *Rhazya stricta* [115] and NF-kB inhibitory is exhibited by the strictamine (**80**) [118]. The total syntheses began with the known chiral aldehyde ester (**74**) to produce enal (**75**) as an important common intermediate. Ultrasound precursor (**76**) was obtained from the enal (**75**) to construct E ring in this work; treatment of the vinyl iodide (**76**) with Pd_2_(dba)_3_/PPh_3_/HCO_2_Na afforded the azabicyclo[3.3.1]nonane ring system. Herein, the role of the ultrasound was crucial to protect the secondary amine as the desired product secondary amine was not very stable and masked as a carbamate under ultrasonic irradiation as a green method employing Boc_2_O and Na_2_CO_3_ at room temperature, affording tetracyclic ester (**77**) in 48% yield over two steps (Scheme 14). Tetracyclic ester (**77**) was effective to produce the known 16-*epi*-strictramine (**78**) in 41% yield over three steps and (–)-rhazinoline (**79**) in 68% yield over two steps. The common crucial intermediate enal (**75**) was also useful to generate another natural product (–)-strictamine (**80**) as the distinctive synthetic strategy.

#### 2.6.2 Deprotection under ultrasonic irradiation

##### 2.6.2.1 Deprotection of -SO_2_Ph under ultrasonic irradiation

###### 2.6.2.1.1 Total synthesis of (-)-haliclonin a

**Scheme 15:**
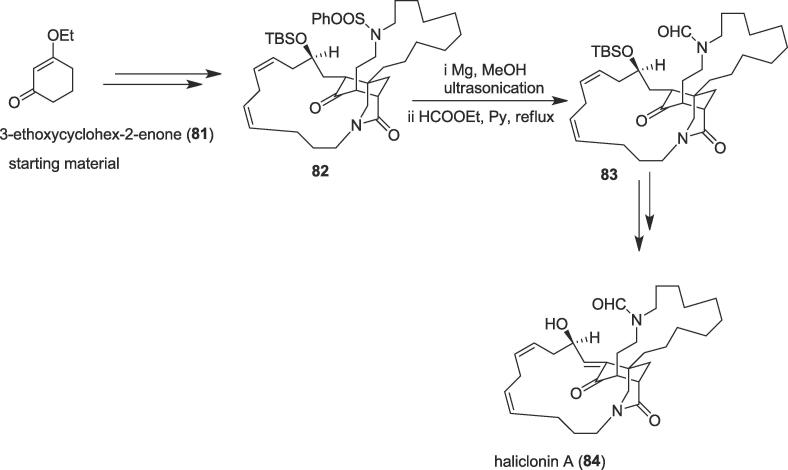
Ultrasound-assisted total synthesis of (-)-haliclonin A.

The first asymmetric total synthesis of the macrocyclic alkaloid (-)-haliclonin A (**84**) was explored by Huang and co-workers in 2016 from 3-ethoxycyclohex-2-enone (**81**) as a starting material using ultrasound irradiation as a non-contaminating tool [119]. The bioactive alkaloid (-)-haliclonin A (**84**) was obtained from a marine sponge *Haliclona* sp. collected from Korean waters in 2009 [120]. This natural (-)-haliclonin A (**84**) has two aza-macrocycles and is structurally linked to sarains A-C, but contains an unprecedented 3-azabicyclononane framework also [121]. Moderate antibacterial activity against several microbial strains and cytotoxic property against the K562 leukemia cell line was exhibited by this unique natural alkaloid [119]. To complete the enantioselective total synthesis of (-)-haliclonin A (**84**), ultrasound precursor tetracyclic diene (13*Z*,16*Z*)- (**82**) was obtained from starting material 3-ethoxycyclohex-2-enone (**81**) over several steps using a novel organocatalytic asymmetric conjugate addition, a Pd-promoted cyclization, a samarium(II) iodide (SmI_2_)-mediated intermolecular reductive coupling, Dess–Martin oxidation and Wittig reaction. Herein, the role of ultrasonic irradiation was crucial to provide key intermediate diamide (**83**) from tetracyclic diene (**82**) *via* a deprotection method. Tetracyclic diene (**82**) was desulfonylated by treatment with Mg in methanol under ultrasonication [122] and the resulting crude amine reacted with ethyl formate/pyridine (HCOOEt/Py) to afford diamide ketone (**83**) through formylation reaction in 82% yield over two steps (Scheme 15). Diamide (**83**) was able to produce the desired natural alkaloid (-)-haliclonin A (**84**) ultimately and the structure of (-)-haliclonin A (**84**) has been confirmed by this total synthesis, and its absolute configuration clarified as 1*E*,3*R*,4*S*,6*R*,11*R*,13*Z*,16*Z*.

##### 2.6.2.2 Deprotection of Tce under ultrasonic irradiation

###### 2.6.2.2.1 Total synthesis of nosiheptide

**Scheme 16:**
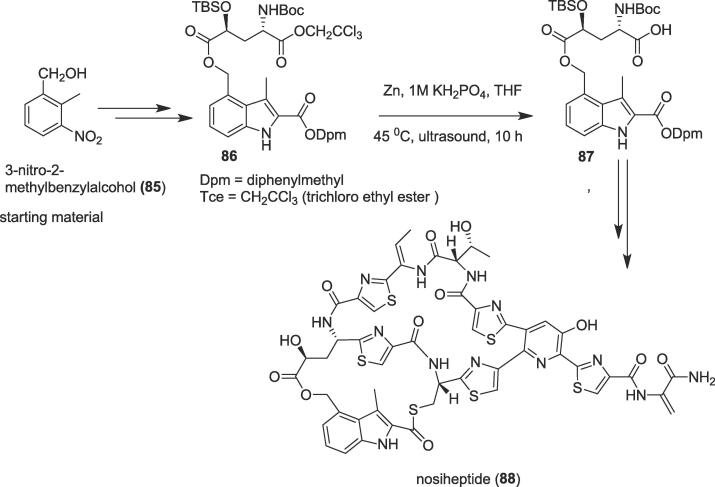
Ultrasound-assisted total synthesis of nosiheptide.

In 2016, Arndt and co-workers explored the total synthesis of the bismacrocyclic thiopeptide antibiotic nosiheptide (**88**) through the reductive deprotection under ultrasound irradiation [123]. Nosiheptide (**88**) exhibits exceptional antibiotic property *in vitro* and a mouse model against critical Gram-positive pathogens such as VRE, MRSA, or Clostridium difficile [124], [125], [126]. Thiopeptide antibiotic nosiheptide (**88**) has a unique 3-hydroxypyridine core, a sterically hindered aromatic B-ring thiolactone, and substituted thiazoles. It also contains an indolylmethyl ester, all embedded within a bismacrocyclic scaffold endowed with a pendant dehydroaminoacid side chain [123]. The total synthesis of this bismacrocyclic thiopeptide antibiotic remains a challenge due to its complex structure and interesting biological activity. The authors commenced the total synthesis of nosiheptide (**88**) from 3-nitro-2-methylbenzylalcohol (**85**) to afford ultrasound precursor trichloro ethyl ester (Tce ester, **86**) over eight steps. Now, Tce ester (**86**) undergoes the reductive deprotection reaction to furnish acid (**87**) through ultrasound irradiation as a green tool (Scheme 16). Treatment of the Tce ester (**86**) with Zn in the presence of the 1 M KH_2_PO_4_ in THF provided acid (**87**) at 45 ^0^C for 10 h in 95% yield under ultrasonication. Finally, acid (**87**) was effective to yield the desired natural product nosiheptide (**88**) through the assembly of a fully functionalized linear precursor followed by consecutive macrocyclizations.

##### 2.6.2.3 Deprotection of trichloroethyloxycarbonyl (Troc) under ultrasonic irradiation

###### 2.6.2.3.1 Total synthesis of enigmazole a

**Scheme 17:**
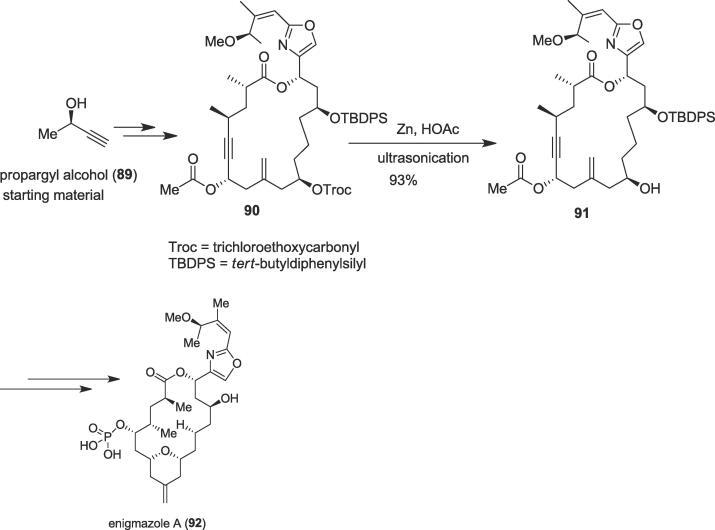
Ultrasound-assisted total synthesis of enigmazole A.

Furstner et al. accomplished an efficient total synthesis of the architecturally complex marine metabolite enigmazole A (**92**) through an ultrasound-accelerated trichloroethyloxycarbonyl (Troc) cleavage in 2016 [127]. Enigmazole A (**92**), a novel phosphate-bearing macrolide, was obtained from a sponge of the genus *Cinachyrella enigmata* collected off the Papua New Guinean coastline [128]. The concise total synthesis of phosphorylated marine macrolide enigmazole A (**92**) was of very interest to the organic synthetic community due to its complex structure bearing densely functionalize 2,4-disubstituted oxazole fragment and interesting biological activity namely anticancer activity [129], [130]. The authors started their total synthesis of this natural macrolide from commercial propargyl alcohol (**89**) to afford the desired ultrasound precursor cycloalkyne (**90**) on a gram scale (single largest batch) at ambient temperature over several steps. Next, an efficient ultrasound-accelerated Troc cleavage took place as a green methodology in the presence of the zinc dust in acetic acid to furnish important alcohol (**91**) in excellent yield (93%) as an adequate substrate for the critical π-acid-catalyzed reaction cascade (Scheme 17). Ultimately, this alcohol (**91**) was able to give natural product enigmazole A (**92**) *via* gold-catalyzed [3], [3]-sigmatropic rearrangements as a key step [131]. The total synthesis of this bioactive enigmazole A (**92**) was robust, concise and convergent, and provides the power of transannular functionalization with more yield (up to 3.3%) than previously reported methods (0.41%).

##### 2.6.2.4 Deprotection of tosyl (Ts) under ultrasonic irradiation

###### 2.6.2.4.1 Total synthesis of (–)-galanthamine

**Scheme 18:**
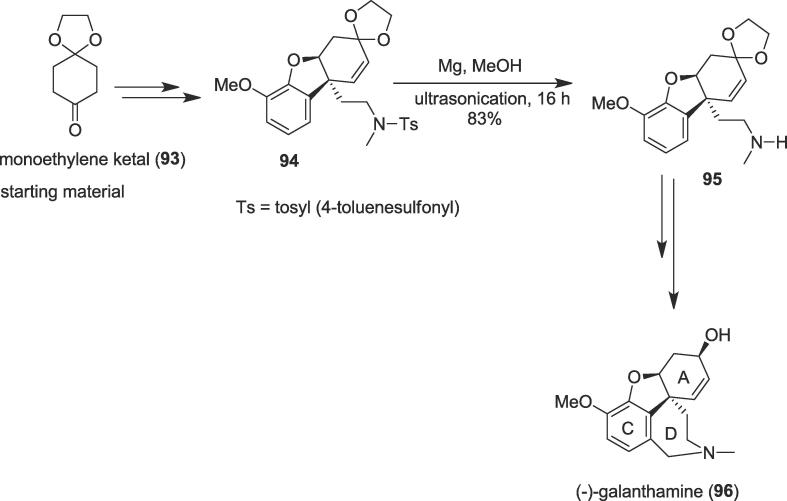
Ultrasound-assisted total synthesis of (–)-galanthamine.

In 2015, Banwell and co-workers achieved a total synthesis of the tetracyclic alkaloid galanthamine (**96**) involving the construction of the seven-membered D-ring through ultrasonic irradiation [132]. The Amaryllidaceae alkaloid (–)-galanthamine (**96**) was obtained from a range of plant sources namely *Galanthus Woronowii* and *Lycoris radia* [133], [134]. This naturally occurring tertiary amine galanthamine (**96**) is used for the treatment of the early stages of Alzheimer’s disease today under its brand names Razadyne among others [135], [136]. The authors began the total synthesis of the (–)-galanthamine (**96**) from the commercially available monoethylene ketal (**93**) of cyclohexane-1,4-dione to construct ultrasound precursor methoxyarene (**94**) that embodies the aromatic C-ring of galanthamine over several steps. Next, treatment of the methoxyarene (**94**) with Mg in methanol provided secondary amine (**95**) in 83% yield under ultrasonication for 16 h as a non-polluting method (Scheme 18). In this crucial stage, the ultrasonic irradiation was very effective to break the tosyl bond for the formation of the seven-membered D-ring of the alkaloid (–)-galanthamine (**96**). Finally, the secondary amine (**95**) yielded the desired bioactive natural product (–)-galanthamine (**96**) over four steps in 83% yield.

###### 2.6.2.4.2 Total syntheses of (–)-kopsanone, and (+)-N-methyl-10,22-dioxokopsane

**Scheme 19:**
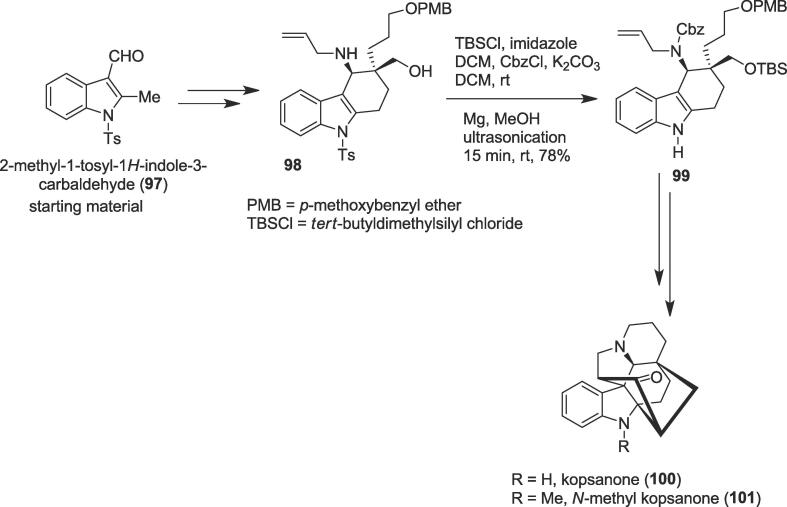
Ultrasound-assisted total syntheses of (–)-kopsanone, and (+)-N-methyl-10,22-dioxokopsane.

In 2020, asymmetric and concise total syntheses of five kopsan alkaloids were disclosed by Ye and co-workers *via* deprotection of the tosyl group under ultrasonic irradiation as a key step [137]. Kopsine the first isolated member of Kopsia indole alkaloids family, as well as many kopsane alkaloids contain an indoline fused to a strained carbocyclic cage [138], [139], [140]. The structure of kopsane alkaloids namely (-)-kopsanone , (+)-kopsanol, (-)-epikopsanol, (+)-10,22-dioxokopsane and (+)-*N*-methyl-10,22-dioxokopsane share a unique heptacyclic skeleton having six stereogenic centers, of which two are vicinal and quaternary [137]. This family of natural products has drawn continuous attention from the synthetic organic and biological community due to their interesting structural pattern and attracting biological activities including cholinergic, antirheumatism, and anti-inflammation effects [141]. Kopsanone (**1 0 0**) was isolated from *Aspidosperma macrocarpon* and it shows monoamine oxidase A (MAO-A) inhibitory activity [142]. The total syntheses of kopsane alkaloids began from the known aldehyde (**97**) to generate ultrasound precursor amino alcohol (**98**) in 76% yield over four steps. Protection of the primary hydroxyl function as its ether of *tert*-butyldimethylsilyl (TBS) and masking the amine group as its benzyloxycarbonyl (Cbz) group derivative followed by cleavage of the *N*-Ts group in the presence of the magnesium powder in methanol (MeOH) furnished secondary amine (**99**) in 62% yield under ultrasonication as a green method at room temperature (Scheme 19). Finally, an amino alcohol (**98**) was very useful to produce natural alkaloids (-)-kopsanone (**1 0 0**) and *N*-methyl kopsanone (**1 0 1**) as well as (+)-kopsanol, (-)-epikopsanol, (+)-10,22-dioxokopsane and (+)-*N*-methyl-10,22-dioxokopsane *via* a PtCl_2_ catalyzed intramolecular [3 + 2] cycloaddition as another crucial step to construct polycyclic indolines with excellent diastereoselectivities.

###### 2.6.2.4.3 Total syntheses of tubulysin U and V

**Scheme 20:**
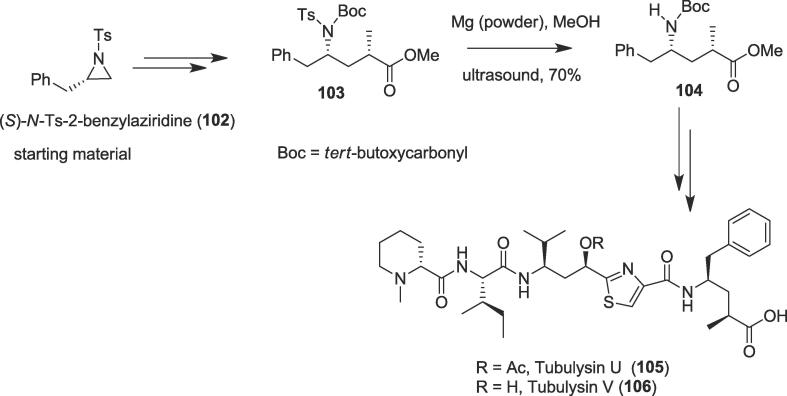
Ultrasound-assisted total syntheses of tubulysin U and V.

A short, stereoselective, and convergent total syntheses of tubulysin U and V was achieved by Wessjohann and co-workers using ultrasound as a green technology [143]. This group planned a total synthesis of tubulysins to reveal the structure–activity relationship for these compounds as tubulysins can be obtained by a fermentation process that yields less than 10 mg L^-1^ [143]. The tubulysins, the most potent anticancer molecules ever discovered from Nature [144], [145], [146], are a family of architecturally complex tetrapeptides which were isolated from the myxobacteria *Archangium gephyra* and *Angiococus disciformis* [147]. Tubulysins are structurally linked to the marine natural product; the complex molecular architecture of it contains four uncommon amino acid fragments, *N*-methylpipecolic acid (Mep), isoleucine (Ile), tubuvaline (Tuv), and tubuphenylalanine (Tup) or Tubutyrosine (Tut) [148]. Biologically, they show potent cytotoxic activities and many members of this family surpass the well-known chemotherapeutic agents such as taxol, epothilones, and vinblastine by a factor of 20–1000 concerning growth inhibition potential [149]. Tubulysins U (**1 0 5**) and V (**1 0 6**), the representative for the basic structures of the highest potent members in this family, tubulysins (**1 0 5**) shows extraordinary potent antiproliferative activity in 1A9 ovarian cancer cells (IC_50_ = 0.65 nM), MCF-7 breast cancer cells (IC_50_ = 0.4 nM) and for *in vitro* inhibition of tubulin polymerization (IC_50_ = 1.9 µM) [150]. The authors commenced the total synthesis of tubulysins U (**1 0 5**) and V (**1 0 6**) from commercial available (*S*)-*N*-Ts-2-benzylaziridine (**1 0 2**) to afford the ultrasound precursor methylphenylsulfonamido ester (**1 0 3**) in four steps. Treatment of the phenyl ester (**1 0 3**) with Mg (powder) in methanol provided secondary amine (**1 0 4**) in a 70% yield under ultrasonication (Scheme 20). The secondary amine (**1 0 4**) was effective to yield the desired products tubulysins U (**1 0 5**) and V (**1 0 6**) finally.

### 2.7 Saponification under ultrasonic irradiation

Saponification is the method in which triglycerides reacted with a strong base (typically sodium or potassium hydroxide) to produce fatty acid metal salts (soaps) during the soap-making process. Plant extracts, such as rosemary, vegetable, and essential oils are often mixed to soaps to increase quality and sensory appeal. Natural soaps are normally obtained from vegetable or plant oils used as soap feedstock and included natural fragrances and/or organic constituents comprised as additives [151], [152].

#### 2.7.1 Total synthesis of hemiasterlin

**Scheme 21:**
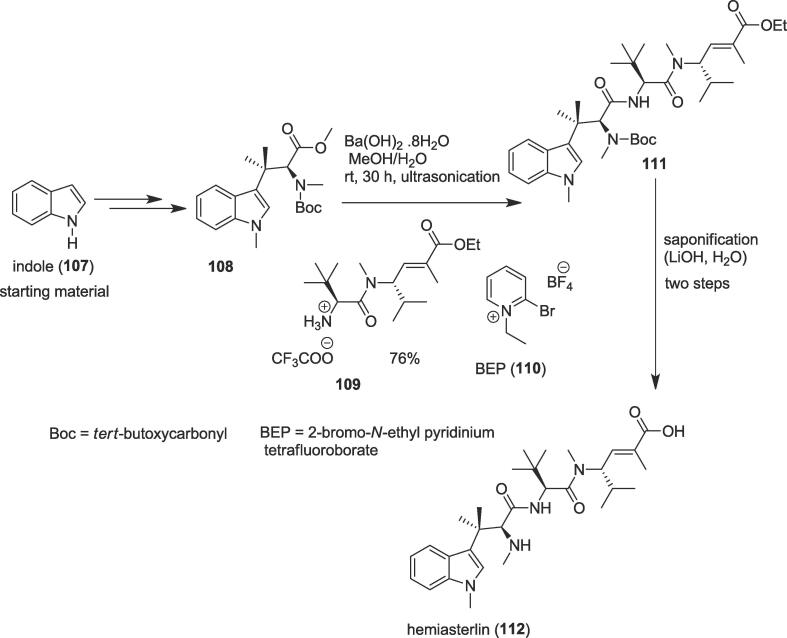
Ultrasound-assisted total synthesis of hemiasterlin.

In 2017, an efficient total synthesis of the marine bioactive natural product hemiasterlin (**1 1 2**) was completed by Lindel et al. involving a crucial saponification reaction under ultrasonic irradiation [153]. The potently cytotoxic marine peptide hemiasterlin (**1 1 2**) was isolated from marine sponges that, like other structurally diverse peptide-like compounds, attaches to the Vinca-peptide site in tubulin, disrupts usual microtubule dynamics, and, at stoichiometric amounts, depolymerizes microtubules [154], [155]. The total synthesis of this cytotoxic tri-peptide hemiasterlin (**1 1 2**) was initiated from indole (**1 0 7**) to produce methyl ester (**1 0 8**) as an ultrasound precursor over several steps. Next, this ester (**1 0 8)** was saponified in the presence of the suspension of barium hydroxide [Ba(OH)_2_·8 H_2_O] in methanol/water by a temperature-controlled ultrasonic bath for 30 h at room temperature as a non-polluting source of energy to furnish carboxylic acid. The generated carboxylic acid coupled efficiently with dipeptide (**1 0 9**) in the presence of 2-bromo-*N*-ethyl pyridinium tetrafluoroborate (BEP) (**1 1 0**) to afford hemiasterlin ethyl ester (**1 1 1**) in 76% yield for only 15 min [156] (Scheme 21). Ethyl ester (**1 1 1**) was again saponified in the presence of the lithium hydroxide (LiOH) and after work-up [156] it provided the desired natural product hemiasterlin (**1 1 2**) in 67% yield over two steps.

## 3 Ultrasound as an unconventional technique

Chemical reactions need tedious and long procedures frequently, which, sometimes, can be avoided by employing ultrasound as an unconventional activation technique to save time, increase selectivities, provide high yields with a purity of the products, and permit us also to explore novel transformation profiles. A large numbers of chemical transformations are available to obey these beneficial qualities of ultrasonic irradiation [15], [16], [17]; few examples are cited in this section as this review tries to concentrate on applications of ultrasound in the total synthesis of bioactive natural products. Thus, the *O*-alkylation of 5-hydroxychromones such as 5-hydroxy-4-oxo-4*H*-1-benzopyran-2-carboxylic acid ethyl ester is a difficult method under conventional protocol due to hydrogen bonding between the carbonyl and OH group [15]. As a result, a low yield (28%) of the targeted *O*-propyl product is obtained at 65 ^0^C for 1.5 h. On the other hand, the role of ultrasound was crucial as a green tool to increase the yield of the desired *O*-propyl product (nearly 100%) under similar conditions. Besides, the chemical transformation can be made more specific using alumina and sonication to deliver more pure products instead of a mixture of products under normal stirred conditions [15]. In this case, benzaldehyde was treated with potassium cyanide and ammonium chloride in acetonitrile afforded to a mixture of products under conventional conditions; the optimum conversion conditions need the presence of suspended alumina along with sonication as an unconventional technique and then the yield of desire aminonitrile attains 90% (Strecker synthesis). The effect of a cavitation becomes more significant in the case of transformations involving solid–liquid phase transfer catalysis [15]. The *N*-alkylation of indole took place with RBr [R = CH_3_(CH_2_)_11_] to deliver *N*-alkylated product a 19% yield in the presence of solid KOH at 25 ^0^C for 3 h using *tert*-butylammonium nitrate under conventional method. The yield of the desired product was substantially enhanced by sonication as an unconventional activation technique to around 90% after only 80 min [15].

## 4 Conclusion

The total synthesis has a privileged position of trust to confirm the hypothetical complex structures of natural products despite sophisticated analytical and spectroscopic instrumentation and techniques are available presently. Moreover, total synthesis is also useful to prepare rare bioactive natural products in the laboratory as several bioactive natural products are obtained in small quantities from natural sources. Bioactive natural products have played a crucial role in the field of drug discovery and they continue to attract intense attention due to their structural arrangement with higher diverse core ring scaffolds and attracting biological and pharmacological activities. The artistic aspect of total synthesis of bioactive natural products continues to be praised today as it may provide environmental protection through the concept of green or clean chemistry namely ultrasonic irradiation is used as a non-polluting tool to synthesize organic molecules elegantly. Green chemistry is related to creativity and the development of innovative research. Ultrasound is a unique technique related to cavitation which is nowadays a well-regarded eco-environmental technology in organic synthesis as well as a total synthesis of bioactive natural products. Ultrasound irradiated reactions differ from conventional energy sources in terms of reaction rates, yields, selectivities, and purity of the products. The use of ultrasound waves as a non-polluting source of energy is of great interest in the field of sustainable and pharmaceutical chemistry. Students and researchers learn how to solve existing problems and develop immunity to disappointment and depression upon failing during the total synthesis of bioactive natural products using ultrasound as a non-polluting source of energy. So the present review will surely make some impact in this direction as it inspires the new generation of practitioners of the art and science of ultrasound in the total synthesis of bioactive natural products to exploit their potential as opportunities to enhance our knowledge in chemical as well as biological and medicinal sciences and apply it to invent new medicines for the community.

## CRediT authorship contribution statement

**Sasadhar Majhi:** Conceptualization, Methodology, Writing &ndash; original draft, Writing &ndash; review &amp; editing.

## Declaration of Competing Interest

The authors declare that they have no known competing financial interests or personal relationships that could have appeared to influence the work reported in this paper.
